# The HGF/Met/NF-κB Pathway Regulates RANKL Expression in Osteoblasts and Bone Marrow Stromal Cells

**DOI:** 10.3390/ijms21217905

**Published:** 2020-10-24

**Authors:** Masanobu Tsubaki, Shiori Seki, Tomoya Takeda, Akiko Chihara, Yuuko Arai, Yuusuke Morii, Motohiro Imano, Takao Satou, Kazunori Shimomura, Shozo Nishida

**Affiliations:** 1 Division of Pharmacotherapy, Kindai University Faculty of Pharmacy, Kowakae, Higashi-Osaka 577-8502, Japan; tsubaki@phar.kindai.ac.jp (M.T.); seki_kindai@yahoo.co.jp (S.S.); takeda@phar.kindai.ac.jp (T.T.); chihara.kindai@gmail.com (A.C.); arai.kindai@gmail.com (Y.A.); morii_kindai@yahoo.co.jp (Y.M.); 2 Department of Pharmacy, Municipal Ikeda Hospital, Ikeda 563-0025, Japan; shimomura_ikeda@yahoo.co.jp; 3 Department of Surgery, Kindai University Faculty of Medicine, Osakasayama, Osaka 589-0014, Japan; imano@med.kindai.ac.jp; 4 Department of Pathology, Kindai University Faculty of Medicine, Osakasayama, Osaka 589-0014, Japan; takaosat@med.kindai.ac.jp

**Keywords:** HGF, RANKL, osteoblast, bone marrow stromal cells, NF-κB, multiple myeloma

## Abstract

Multiple myeloma (MM)-induced bone disease occurs through hyperactivation of osteoclasts by several factors secreted by MM cells. MM cell-secreted factors induce osteoclast differentiation and activation via direct and indirect actions including enhanced expression of receptor activator of nuclear factor κB ligand (RANKL) in osteoblasts and bone marrow stromal cells (BMSCs). Hepatocyte growth factor (HGF) is elevated in MM patients and is associated with MM-induced bone disease, although the mechanism by which HGF promotes bone disease remains unclear. In the present study, we demonstrated that HGF induces RANKL expression in osteoblasts and BMSCs, and investigated the mechanism of induction. We found that HGF and MM cell supernatants induced RANKL expression in ST2 cells, MC3T3-E1 cells, and mouse BMSCs. In addition, HGF increased phosphorylation of Met and nuclear factor κB (NF-κB) in ST2 cells, MC3T3-E1 cells, or mouse BMSCs. Moreover, Met and NF-κB inhibitors suppressed HGF-induced RANKL expression in ST2 cells, MC3T3-E1 cells, and mouse BMSCs. These results indicated that HGF promotes RANKL expression in osteoblasts and BMSCs via the Met/NF-κB signaling pathway, and Met and NF-κB inhibitors suppressed HGF-induced RANKL expression. Our findings suggest that Met and NF-κB inhibitors are potentially useful in mitigating MM-induced bone disease in patients expressing high levels of HGF.

## 1. Introduction

MM is the second most common hematologic malignancy and is characterized by clonal proliferation of malignant plasma cells within bone marrow. MM accounts for 1% of new cancers worldwide and has a five-year survival rate of 49% [1]. About 80–90% of MM patients develop bone disease, leading to a 16-fold increase in their risk of skeletal fractures, which markedly reduces their quality of life [1,2]. Currently, bisphosphonates are recommended for treatment of MM-induced bone disease. Bisphosphonates inhibit osteoclast formation and induce apoptosis in osteoclasts and tumor cells [3,4,5,6,7,8]. However, they can produce adverse effects such as jaw osteonecrosis and renal impairment.

MM-induced bone disease proceeds because of interactions between MM cells and the bone marrow microenvironment, which promote osteoclastic bone resorption and bone loss. MM cells cause an imbalance in the normal bone remodeling process by secreting soluble factors that directly or indirectly activate osteoclasts [9]. Additionally, Dickkopf-1 and soluble Frizzled related protein 2 secreted by the MM cells inhibited osteoblast maturation of the bone marrow stromal cells via inhibition of Wnt signaling. They also secreted interferon γ and tumor necrosis factor α (TNF-α), which induce osteoblast apoptosis [10], thereby leading to depletion of osteoblast function and bone loss. Normal bone remodeling is regulated by the ratio of osteoprotegerin to receptor activator of nuclear factor κB (RANK) and its ligand (RANKL), which induce osteoclast differentiation and activation [11,12]. RANKL, a gene product expressed in osteoblasts and BMSCs, is a necessary factor for differentiation of osteoclast precursor cells to mature osteoclasts and for promotion of osteoclast survival [6,13]. MM cells secrete interleukin-6 (IL-6), TNFα, and macrophage inflammatory protein-1α (MIP-1α), which induce RANKL expression in osteoblasts and BMSCs and directly promote osteoclast differentiation [9,14,15,16,17,18,19,20]. Some studies have indicated that these factors are increased in the plasma of MM patients compared to that of healthy donors [21,22]. In other studies, these factors are not elevated among MM patients [23], suggesting that other factors may contribute to MM-induced bone disease in patients without increased levels of these factors.

HGF promotes cell proliferation, survival, and migration by activating its receptor tyrosine kinase Met [24]. HGF is secreted by MM cells isolated from patients and cells of MM cell lines, and HGF levels are elevated in the plasma of MM patients [25]. Overexpression of Met in MM patients has been correlated with poorer prognosis compared to that of MM patients with low expression of Met [26]. HGF acting in concert with RANKL has been shown to promote osteoclast differentiation, while HGF acting alone did not affect osteoclast formation and suppressed osteoblastogenesis, thereby contributing to MM-induced bone disease [27,28]. However, the potential effect of induction of RANKL by HGF is not well-defined. In the present study, we demonstrated that HGF induces RANKL expression in osteoblasts and BMSCs and investigated the mechanism.

## 2. Results

### 2.1. Expression of HGF Was Increased in Patients with MM Cells and Bone Lytic Lesions in MM Patients

**Figure 1:**
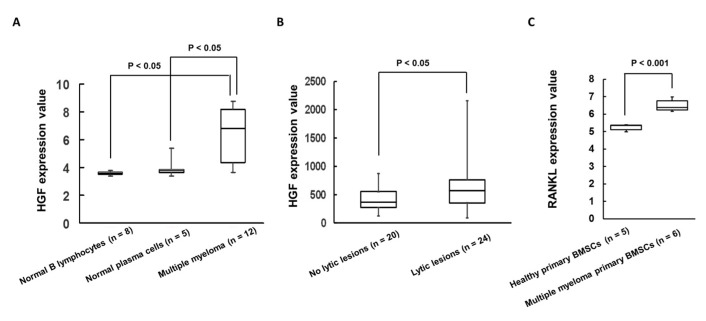
(**A**,**B**) The expression of HGF was analyzed using the (**A**) GSE6691 and (**B**) GSE755 dataset. (**C**) The expression of RANKL was evaluated using the GSE108915 and GSE78235 datasets.

First, we analyzed HGF expression using Gene Expression Omnibus (GEO) dataset GSE6691 including normal B lymphocytes or normal plasma cells from healthy donors and patients with MM cells, and GSE755 including non-lytic bone lesions and lytic bone lesions in patients with MM. We found that expression of HGF was significantly increased in patients with MM cells and bone lytic lesions in MM patients (Figure 1A,B). Additionally, RANKL and HGF expression levels were found to be higher in BMSCs derived from MM patients than in normal individuals (Figure 1C).

### 2.2. HGF Promotes Expression of RANKL in ST2 Cells, MC3T3-E1 Cells, and Mouse BMSCs

**Figure 2:**
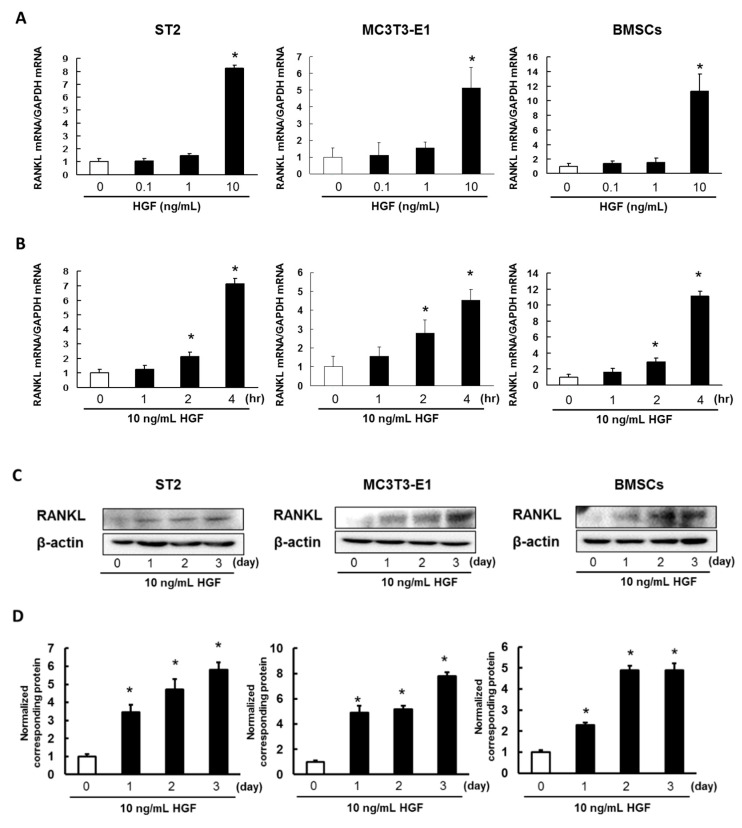
HGF enhances RANKL expression in BMSCs and osteoblasts. (**A**) ST2 cells, MC3T3-E1 cells, and mouse BMSCs were administrated with 0.1, 1, or 10 ng/mL HGF for 4 h. (**B**) ST2 cells, MC3T3-E1 cells, and mouse BMSCs were administrated with 10 ng/mL HGF for 1, 2, or 4 h. Total RNA was extracted, and the RANKL mRNA expression was analyzed by real-time PCR. The results are representative of five independent experiments. * *p* < 0.01, as compared to controls (one-way ANOVA with Dunnett’s test). (**C**) ST2 cells, MC3T3-E1 cells, and mouse BMSCs were incubated with 10 ng/mL HGF. At various time points, the cell lysates were analyzed by western blotting using anti-RANKL antibody. (**D**) Quantification of the amount of RANKL, normalized to the amounts of β-actin. The results are representative of five independent experiments. * *p* < 0.01, compared to controls (one-way ANOVA with Dunnett’s test).

We investigated whether HGF promotes expression of RANKL mRNA in ST2 cells, MC3T3-E1 cells, and mouse BMSCs. RANKL mRNA expression increased in ST2 cells, MC3T3-E1 cells, and mouse BMSCs when treated with 10 ng/mL HGF (Figure 2A). In the experiments described below, cells were treated with HGF at a concentration of 10 ng/mL.

We also investigated how RANKL mRNA expression changed over time after adding HGF to ST2 cells, MC3T3-E1 cells, and mouse BMSCs. RANKL mRNA expression began to increase at 2 h after the addition of HGF (Figure 2B).

Next, we examined RANKL protein expression in HGF-treated ST2 cells, MC3T3-E1 cells, and mouse BMSCs. Stimulation of ST2 cells, MC3T3-E1 cells, and mouse BMSCs with HGF increased RANKL protein expression in a time-dependent manner (Figure 2C,D).

### 2.3. Enhancing the Expression of RANKL in ST2 Cells, MC3T3-E1 Cells, and Mouse BMSCs Using the Culture Media Collected from Multiple Myeloma Cells

**Figure 3:**
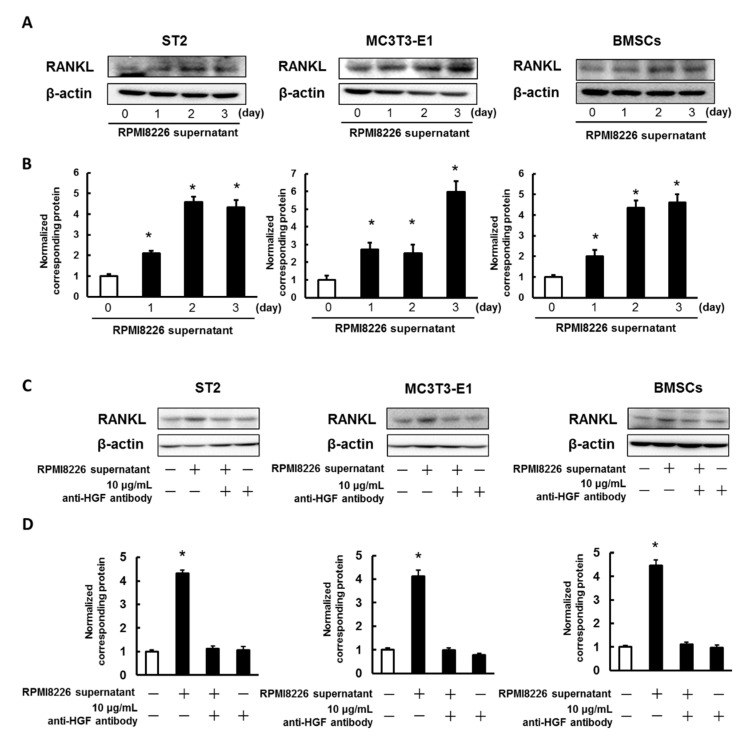
RPMI8226 cell supernatant induces RANKL expression. (**A**) ST2 cells, MC3T3-E1 cells, and mouse BMSCs were incubated with RPMI8226 cell supernatant. At various time points, the cell lysates were analyzed by western blotting using anti-RANKL antibody. (**B**) Quantification of the amount of RANKL, normalized to the amounts of β-actin. The results are representative of five independent experiments. * *p* < 0.01, compared to controls (one-way ANOVA with Dunnett’s test). (**C**) ST2 cells, MC3T3-E1 cells, and mouse BMSCs were incubated with RPMI8226 cell supernatant and 10 μg/mL anti-HGF neutralizing antibody (R&D Systems, Minneapolis, Minnesota, USA). After incubation for 72 h, the cell lysates were analyzed through western blot analysis using the anti-RANKL antibody. (**D**) Quantification of the level of RANKL was performed by normalizing it to the β-actin level. The results of five independent experiments are presented here. * *p* < 0.01, when compared to the controls (one-way ANOVA with Dunnett’s test).

Increased RANKL expression was observed in ST2 cells, MC3T3-E1 cells, and mouse BMSCs treated with culture media from RPMI8226 cells, which was found to contain HGF (2950 ± 355 pg/mL) and very low levels of MIP-1α, TNF-α, IL-6, IL-1β, and basic fibroblast growth factor (bFGF) expression (Figure 3A,B and Figure S1, Supplementary Materials). Additionally, the anti-HGF neutralizing antibody suppressed RPMI8226 supernatant-induced RANKL expression in ST2 and MC3T3-E1 cells, and mouse BMSCs (Figure 3C,D).

### 2.4. HGF Activated the Met/NF-κB Pathway in ST2 Cells, MC3T3-E1 Cells, and Mouse BMSCs

**Figure 4:**
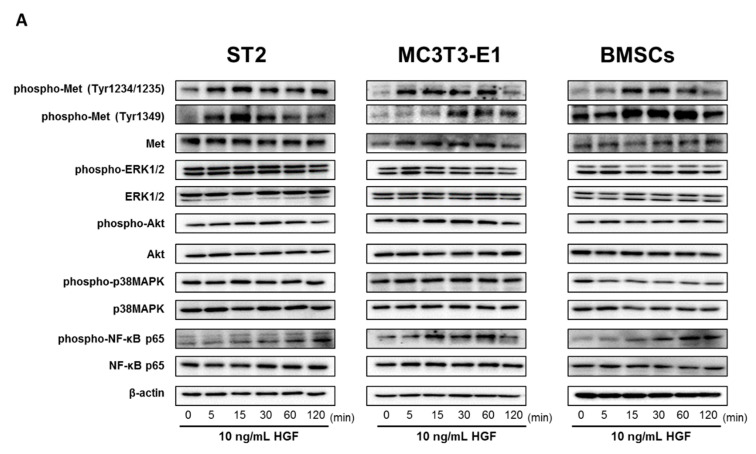

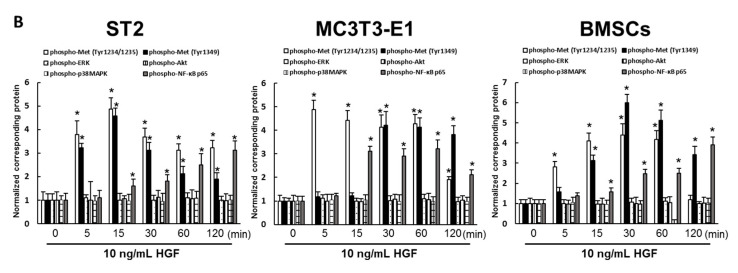
HGF induces the activation of the Met/NF-κB pathway in BMSCs and osteoblasts. (**A**) ST2 cells, MC3T3-E1 cells, and mouse BMSCs were incubated with 10 ng/mL HGF. At various time points, cell lysates were examined by western blotting assay using indicated antibodies. (**B**) Quantification of protein expression, and the normalized corresponding protein, respectively. The results are representative of five independent experiments. * *p* < 0.01, compared to controls (one-way ANOVA with Dunnett’s test).

Treatment of ST2 cells with HGF increased phosphorylated Met (Tyr1234/1235) and Met (Tyr1349) levels at time points from 5 to 120 min after the addition of HGF (Figure 4). The amount of phosphorylated NF-κB p65 increased at times ranging from 15 to 120 min after HGF stimulation. However, stimulation with HGF did not affect levels of extracellular regulated protein kinase 1/2 (ERK1/2), Akt, or p38 mitogen activated protein kinase (p38MAPK) phosphorylation (Figure 4 and Figure S2, Supplementary Materials).

Treatment of MC3T3-E1 cells with HGF induced phosphorylation of Met (Tyr1234/1235) at 5–120 min. Phosphorylation of Met (Tyr1349) was transiently enhanced at 30–120 min after treatment with HGF (Figure 4). Phosphorylation of NF-κB p65 increased at 15–120 min after HGF stimulation. However, stimulation with HGF did not affect levels of ERK1/2, Akt, or p38MAPK phosphorylation (Figure 4 and Figure S2, Supplementary Materials).

Treatment of mouse BMSCs with HGF induced phosphorylation of Met (Tyr1234/1235) at 5–60 min. Phosphorylation of Met (Tyr1349) was transiently enhanced at 15–120 min after treatment with HGF (Figure 4). Phosphorylation of NF-κB p65 increased at 15–120 min after HGF stimulation. However, stimulation with HGF did not affect levels of ERK1/2, Akt, or p38MAPK phosphorylation (Figure 4 and Figure S2, Supplementary Materials).

### 2.5. Met and NF-κB Inhibitors Suppressed HGF-Induced RANKL Expression

The results described thus far indicate that HGF induces RANKL expression via the Met/NF-κB pathway. The Met inhibitors have previously been reported to suppress myeloma-induced bone loss in tumor-bearing mice [29]. It has also been demonstrated that bortezomib suppresses RANKL-induced NF-κB activation in human osteoclasts and induces bone resorption markers in patients with MM [30]. Moreover, in our previous studies, we demonstrated that dimethyl fumarate (DMF) suppresses NF-κB nuclear translocation in various cells [31,32,33]. Based on these findings, we used tepotinib, a Met inhibitor, bortezomib, a proteasome inhibitor, and DMF, an NF-κB inhibitor in the present study. To confirm this hypothesis, ST2 cells, MC3T3-E1 cells, and mouse BMSCs were treated with tepotinib, bortezomib, or DMF, in order to evaluate whether inhibiting Met and/or NF-κB activation suppresses RANKL expression at mRNA and protein levels.

**Figure 5:**
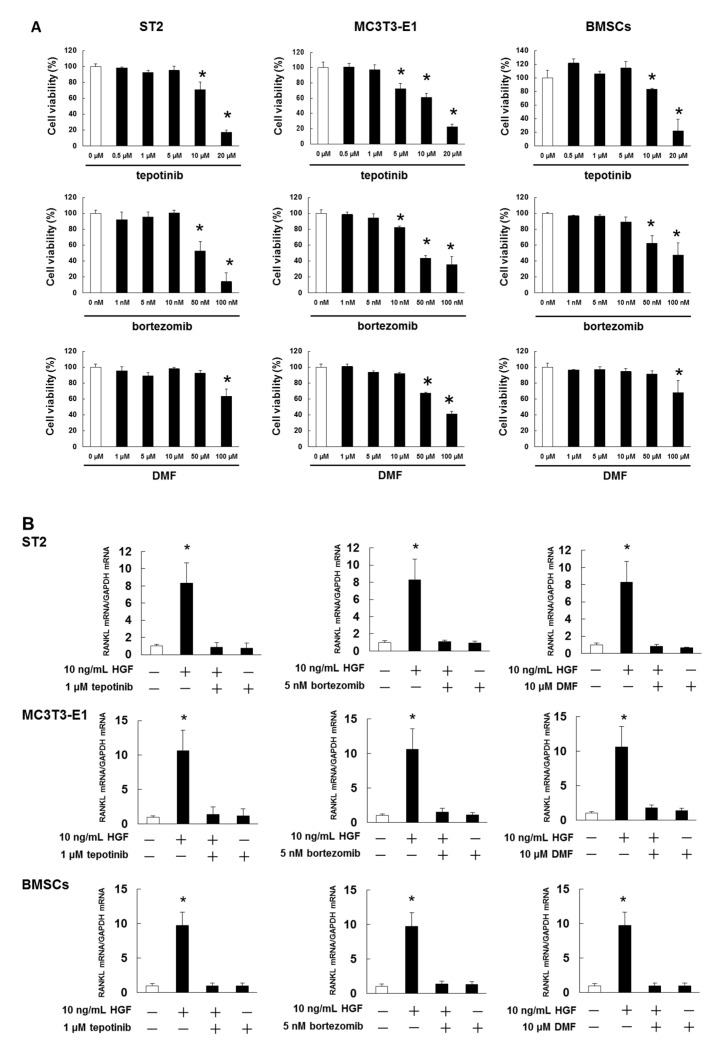

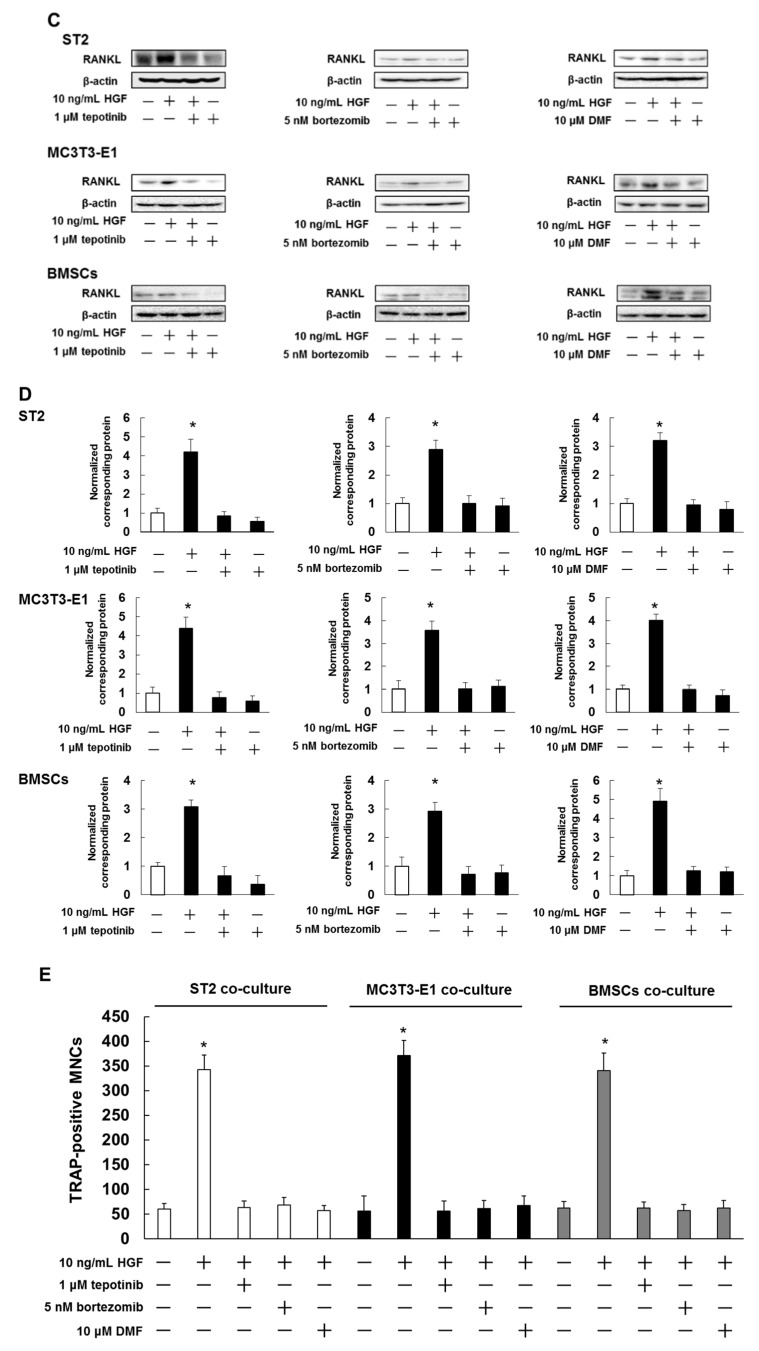
Met and NF-κB inhibitors suppress HGF-induced RANKL expression. (**A**) ST2 cells, MC3T3-E1 cells, and mouse BMSCs were incubated in 96-well plates for 24 h and then treated with various concentrations of tepotinib, bortezomib, or DMF. After 5 days, cell viability was quantified by the trypan blue dye assays. The results are representative of five independent experiments. * *p* < 0.01 vs. the controls (one-way ANOVA with Dunnet’s test). (**B**–**D**) ST2 cells, MC3T3-E1 cells, and mouse BMSCs were treated with 1 μM tepotinib, 5 nM bortezomib, or 10 μM DMF. After incubation for 72 h, HGF was added to give the final concentration of 10 ng/mL. (**B**) After incubation for 4 h, RNA was extracted, and RANKL mRNA expression was analyzed by real time PCR. The results are representative of five independent experiments. * *p* < 0.01, as compared to controls (one-way ANOVA with Dunnett’s test). (**C**) After incubation for 72 h, the cell lysates were analyzed by western blotting using anti-RANKL antibody. (**D**) Quantification of the amount of RANKL, normalized to the amounts of β-actin. The results are representative of five independent experiments. * *p* < 0.01, compared to controls (one-way ANOVA with Dunnett’s test). (**E**) After culturing ST2 cells, MC3T3-E1 cells, and mouse BMSCs, the cells were individually co-cultured with RAW264.7 cells and tartrate-resistant acid phosphatase (TRAP)-positive multinucleated cells (three or more nuclei), and were subsequently counted under a microscope after 7 days. The results of five independent experiments are presented here.

To examine the cytotoxic effects of tepotinib, bortezomib, and DMF on ST2 cells, MC3T3-E1 cells, and mouse BMSCs, cell viability was assessed by treatment with tepotinib at 0.5–20 μM, bortezomib at 1–100 nM, and DMF at 1–100 μM. We determined the cell survival rate, which was defined as the ratio of the number of living cells after 5 days of exposure to various concentrations of these agents to the number of live control cells treated with 0.1% dimethyl sulfoxide (DMSO). Tepotinib at concentrations of 5, 10, or 20 μM; bortezomib at concentrations of 10, 50, or 100 nM; and DMF at concentrations of 50 or 100 μM induced cell death in ST2 cells, MC3T3-E1 cells, and mouse BMSCs (Figure 5A). On the basis of these results, we determined that treatment with 1 μM tepotinib, 5 nM bortezomib, and 10 μM DMF was not cytotoxic to ST2 cells, MC3T3-E1 cells, or mouse BMSCs.

Next, we investigated whether tepotinib, bortezomib, and DMF would inhibit HGF-induced expression of RANKL mRNA and secreted protein. We found that tepotinib, bortezomib, and DMF markedly suppressed the expression of RANKL at both mRNA and protein levels at concentrations that suppressed HGF-induced NF-κB activation (Figure 5B–D and Figure S3, Supplementary Material).

Additionally, we examined whether the inhibition of HGF-induced RANKL expression upon treatment with tepotinib, bortezomib, and DMF suppressed osteoclast formation in a co-culture of RAW264.7 cells with ST2 cells, MC3T3-E1 cells, or mouse BMSCs. HGF treatment promoted osteoclast formation, while treatment with tepotinib, bortezomib, and DMF inhibited osteoclast formation in co-cultures of RAW264.7 cells with ST2 cells, MC3T3-E1 cells, or mouse BMSCs (Figure 5E).

## 3. Discussion

In the present study, we demonstrated that HGF promotes RANKL mRNA and protein expression in ST2 cells, MC3T3E-1 cells, and mouse BMSCs. It has been reported that HGF, vascular endothelial growth factor (VEGF), placental growth factor, and FLT3 ligand support osteoclastogenesis in the absence of macrophage-colony stimulating factor (M-CSF), and VEGF and HGF, when added to M-CSF/RANKL-treated monocyte cultures, induced formation of giant, hyper-multinucleated, and hyper-resorptive osteoclasts [27,34,35,36]. These previous reports also indicated that HGF inhibited bone morphogenetic protein-induced osteoblastogenesis by suppressing activation of Smads, yielding a negative correlation between HGF and bone-specific alkaline phosphatase in the sera of patients with MM [28]. These findings suggest that HGF promotes osteoclast formation via direct action on osteoclast progenitors and enhancement of RANKL expression in BMSCs and osteoblasts.

Elevated levels of plasma HGF in MM patients have been found to correlate with lytic bone disease. In these studies, mean plasma HGF levels were 465.3–648 pg/mL in healthy controls and 2174–2356 pg/mL in patients with MM [37,38]. In addition, plasma HGF levels in MM patients with advanced stage disease were 2833–3157 pg/mL [39]. In this study, we found that the culture media collected from RPMI8226 cells (with HGF concentration of 2950 ± 355 pg/mL, but not very low levels of MIP-1α, TNFα, IL-6, IL-1β, and bFGF) promoted RANKL expression in ST2 cells, MC3T3-E1 cells, and mouse BMSCs and anti-HGF neutralizing antibody suppressed RPMI8226 supernatant-induced RANKL expression. In addition, expression of HGF in MM patients was increased compared to normal B lymphocytes and plasma cells from healthy donors, and HGF expression in lytic bone lesions was higher than that in non-lytic bone lesion patients with MM. Moreover, we found that BMSCs derived from MM patients exhibited higher expression of RANKL than those derived from healthy donors. It is known that MIP-1α, TNFα, IL-6, and bFGF induce RANKL expression in osteoblasts and BMSCs [14,40,41]. It has been indicated that secreted MIP-1α, TNFα, IL-6, and bFGF are present at very low levels in RPMI8226 cells [17,18,20,42]. These findings suggest that high-level secretion of HGF by MM cells promotes RANKL expression, thereby contributing to the induction of bone lytic disease.

Met, an HGF receptor, regulates cell proliferation, survival, and migration. HGF induces Met dimerization and phosphorylation of tyrosine residues in its kinase domain (Y1234 and Y1235) [43]. This triggers activation of signaling molecules including ERK1/2, Akt, p38MAPK, and NF-κB [43,44]. We showed that HGF activates Met and NF-κB, but does not affect ERK1/2, Akt, or p38MAPK in ST2 cells, MC3T3-E1 cells, and mouse BMSCs. In addition, tepotinib, a Met inhibitor, bortezomib, a proteasome inhibitor, and DMF, an NF-κB inhibitor, all suppressed HGF-induced RANKL mRNA and protein expression via suppression of the Met/NF-κB signaling pathway. Moreover, tepotinib, bortezomib, and DMF suppressed osteoclast formation via HGF-induced RANKL expression in co-cultures of RAW264.7 cells with ST2 cells, MC3T3-E1 cells, and mouse BMSCs. It has been shown that the ubiquitin-proteasome pathway plays an important role in regulating and controlling bone metabolism, and that this pathway’s inhibitors, such as bortezomib and carfilzomib, directly suppress osteoclast formation and bone resorption in vitro, and suppress parathyroid hormone-induced RANKL expression via inhibition of NF-κB activation in osteoblasts [45,46,47,48]. Studies have also indicated that bortezomib improved bone disease symptoms in patients with MM [49,50]. Moreover, activation of NF-κB promotes expression of RANKL in human osteoblast-like cells [51]. Tivantinib, a Met inhibitor, reduced tumor burden and inhibited myeloma-induced bone disease in vivo; a Met antagonist inhibited HGF-induced MM cell proliferation, survival, migration, and adhesion, and reversed HGF-induced inhibition of osteoblastogenesis [29,52]. These findings indicate that Met and NF-κB inhibitors inhibit RANKL expression via suppression of the HGF/Met/NF-κB signaling pathway.

It has been reported that HGF promotes RANKL expression via IL-11 secretion in the human osteosarcoma cell line, Saos-2 [53]. Additionally, in this previous study, they showed that the treatment with HGF for 24 h induced IL-11 secretion and RANKL mRNA expression in Saos-2 cells [53]. It has also been shown that the induction of IL-11 mRNA expression plays an essential role in AP-1 activation, and U0126 suppresses IL-11 mRNA expression via inhibition of the MEK/ERK pathway in murine primary osteoblasts, ST2 cells, and Saos-2 cells [54,55]. Moreover, treatment with parathyroid hormone for 2 h induced IL-11 mRNA expression in murine primary osteoblasts, ST2 cells, and Saos-2 cells [54,55]. However, our results clearly indicate that treatment with HGF increased RANKL mRNA expression in 2 h and NF-κB activation in 1 h, but not ERK1/2 activation. Further, the administration of Met and NF-κB inhibitors suppressed HGF-induced RANKL expression after treatment with HGF for 4 h. These findings suggest that the activation of the HGF/Met/NF-κB pathway directly induces RANKL expression in BMSCs and osteoblast cells.

## 4. Materials and Methods

### 4.1. Cell Culture

ST2 cells of a mouse bone marrow stromal cell line and MC3T3-E1 cells of a mouse osteoblast cell line were obtained were from Riken Cell Bank (Ibaraki, Japan). RPMI8226 cells were obtained from Health Science Research Resources Bank (Osaka, Japan). These cells were cultured in RPMI1640 medium (Sigma, St Louis, MO, USA) supplemented with 10% fetal bovine serum (Gibco, Carlsbad, CA, USA), 100 μg/mL penicillin (Gibco), 100 U/mL streptomycin (Gibco), and 25 mM 4-(2-hydroxyethyl)-1-piperazineethanesulfonic acid (pH = 7.4; FUJIFILM Wako, Osaka, Japan) in an atmosphere containing 5% CO_2_. RAW264.7 cells were purchased from DS Pharma Biomedical (Osaka, Japan) and cultured in α-MEM supplemented with 10% FCS, 100 μg/mL penicillin, and 100 U/mL streptomycin in the presence of 5% CO_2_.

Mouse BMSCs were isolated from female C57BL/6J mice (age: 8 weeks; Shimizu Laboratory Animals, Kyoto, Japan) and cultured using standard protocols [56,57]. Animal experiments were approved by the Committee on Laboratory Animal Research of Kindai University.

### 4.2. Quantitative Real-Time Polymerase Chain Reaction (PCR)

The effect of HGF (PeproTech, London, UK) on RANKL expression in ST2 cells, MC3T3-E1 cells, or mouse BMSCs was analyzed by real time PCR as previously described [12].

### 4.3. Western Blotting

Cell lysates were extracted with a lysis buffer (20 mM Tris-HCl (pH = 8.0; FUJIFILM Wako), 150 mM NaCl (FUJIFILM Wako), 2 mM EDTA (FUJIFILM Wako), 100 mM NaF (FUJIFILM Wako), 1% NP40 (FUJIFILM Wako), 1 μg/mL leupeptin (Sigma), 1 μg/mL antipain (Sigma), and 1 mM phenylmethylsulfonyl fluoride (Sigma)). Phosphorylated protein and total protein in these lysates were examined by western blotting assay by using following primary antibodies: anti-phospho-Met (Tyr1234/1235) antibody, anti-phospho-Met antibody (Tyr1349), anti-Met antibody, anti-phospho-ERK1/2 antibody, anti-ERK1/2 antibody, anti-phospho-Akt antibody, anti-Akt antibody, anti-phospho-p38MAPK antibody, anti-p38MAPK antibody, anti-phospho-NF-κB antibody, anti-NF-κB antibody (Cell Signaling Technology, Beverly, MA, USA), anti-RANKL antibody (Santa Cruz Biotechnology, Santa Cruz, CA, USA), and anti-β-actin antibody (Sigma). Proteins were visualized using Luminata Forte (Merck Millipore, Nottingham, UK) according to the manufacturer’s instructions.

### 4.4. Trypan Blue Dye Exclusion Assay

The effect of tepotinib (SelleckChem, Houston, TX, USA), bortezomib (SelleckChem), and DMF (FUJIFILM Wako) on cell survival was determined using the trypan blue dye exclusion assay as previously described [44].

### 4.5. Microarray Dataset Resources

The gene expression profiles of the microarray datasets with the accession numbers GSE6691, GSE755, GSE108915, and GSE78235 were obtained from the National Center of Biotechnology Information (NCBI) GEO database (GEO, http://www.ncbi.nlm.nih.gov/geo/). Expression profile of the HGF gene in B lymphocytes and plasma cells of normal individuals and MM cells was analyzed using GSE6691, and non-lytic lesions and lytic lesions of patients with MM were analyzed using GSE755. Expression profile of RANKL in BMSCs derived from MM patients and normal individuals was analyzed using GSE108915 and GSE78235.

### 4.6. TRAP Staining

The effect of tepotinib, bortezomib, and DMF on osteoclast differentiation via HGF-induced RANKL expression was assessed through TRAP staining as described previously [14].

### 4.7. Statistical Analysis

All the results are expressed as the mean ± S.D. of several independent experiments. Multiple comparisons of the data were performed using ANOVA with Dunnet’s test. *p* values less than 5% were regarded as significant.

## 5. Conclusions

Our results show that HGF induced RANKL expression in osteoblasts and bone marrow stromal cells via the Met/NF-κB signaling pathway, and Met and NF-κB inhibitors suppressed HGF-induced RANKL expression. Although previous studies have discussed the efficacy of MM treatment with Met and/or NF-κB inhibitors, this study demonstrates a clear application for Met and NF-κB inhibitors in treating HGF-related bone disease in MM patients.

## Supplementary Materials

The following information can be found at https://www.mdpi.com/1422-0067/21/21/7905/s1. Figure S1: Secretion of HGF in RPMI8226 cells, Figure S2: Expression of ERK, Akt, p38MAPK, and NF-κB phosphorylation by Luminex assay, Figure S3: Effect of tepotinib, bortezomib, and DMF on HGF-induced NF-κB activation.

## Abbreviations

MM	Multiple myeloma
RANKL	Receptor activator of nuclear factor κB ligand
BMSCs	Bone marrow stromal cells
HGF	Hepatocyte growth factor
NF-κB	Nuclear factor κB
RANK	Receptor activator of nuclear factor κB
IL-6	Interleukin-6
TNFα	Tumor necrosis factor alpha
MIP-1α	Macrophage inflammatory protein-1α
bFGF	Basic fibroblast growth factor
ERK1/2	Extracellular regulated protein kinase 1/2
p38MAPK	p38 mitogen activated protein kinase
M-CSF	Macrophage-colony stimulating factor
VEGF	Vascular endothelial growth factor
