# Supplementary material for: The HGF/Met/NF-κB Pathway Regulates RANKL Expression in Osteoblasts and Bone Marrow Stromal Cells
Source: Int J Mol Sci. 2020 Oct 24;21(21):7905. doi: 10.3390/ijms21217905 (PMC7663721; doi:10.3390/ijms21217905)
Supplement: Supplementary file 1 [file ijms-21-07905-s001.pdf]

Supplementary Figure

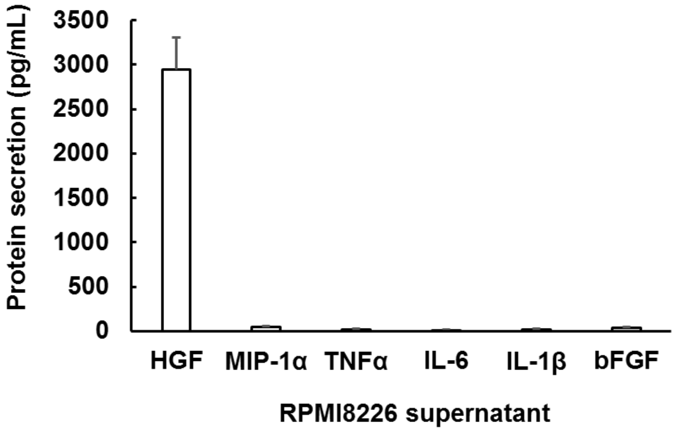

Figure S1. Secretion of HGF in RPMI8226 cells. Supernatants were collected after 48 hours in culture and analyzed for HGF, MIP-1α, TNFα, IL-6, IL-1β, and bFGF by ELISA.

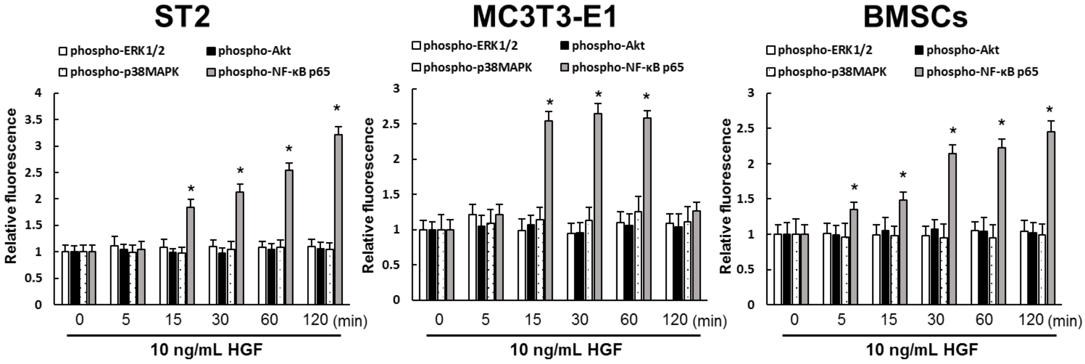

Figure S2. Expression of ERK, Akt, p38MAPK, and NF-κB phosphorylation by Luminex assay. Cells were lysed and phosphorylation of ERK1/2, p38MAPK, Akt, and NF-κB were measured by Luminex assay.

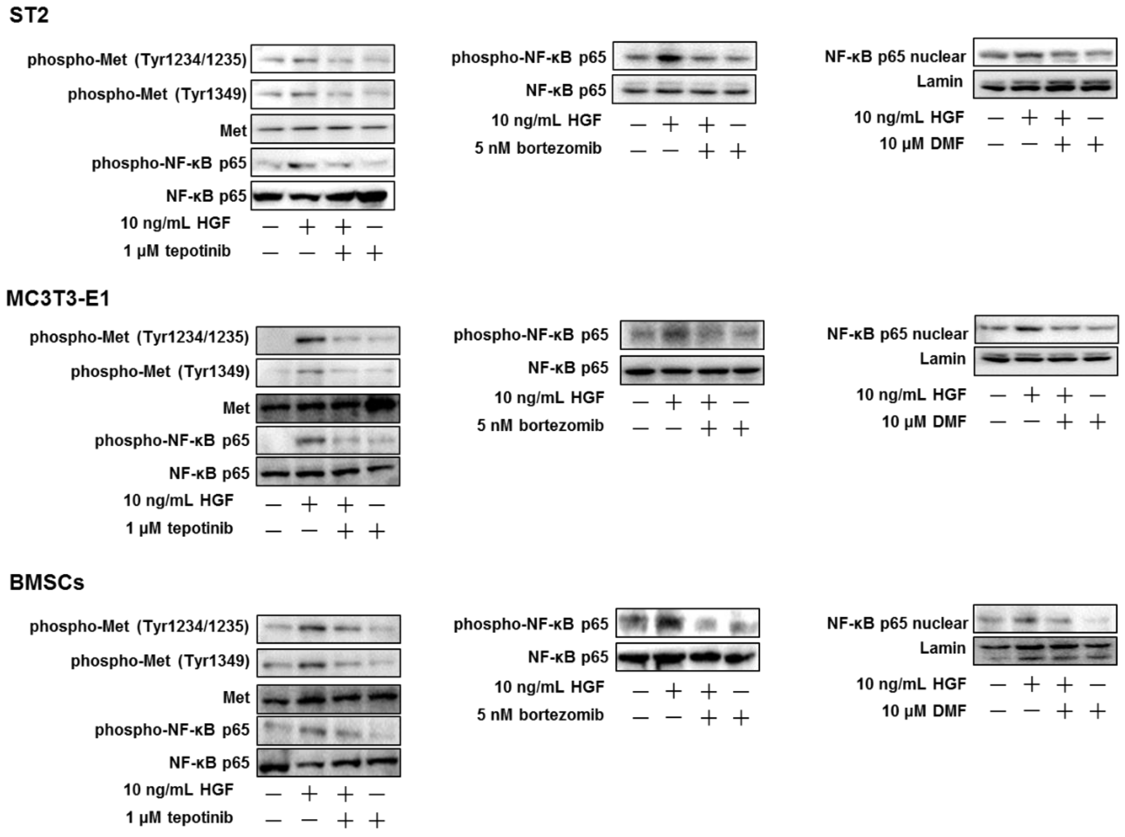

Figure S3. Effect of tepotinib, bortezomib, and DMF on HGF-induced NF-κB activation. ST2 cells, MC3T3-E1 cells, and mouse BMSCs were treated with 1 μM tepotinib, 5 nM bortezomib, or 10 μM DMF. After incubation for 72 h, HGF was added to give the final concentration of 10 ng/mL. After incubation for 72 h, the cell lysates were analyzed by western blotting using anti-phospho-Met (Tyr1234/1235) antibody, anti-phospho-Met (Tyr1349) antibody, anti-Met antibody, anti-phospho-NF-κB antibody, anti-NF-κB antibody, and anti-Lamin antibody.

Figure 1C

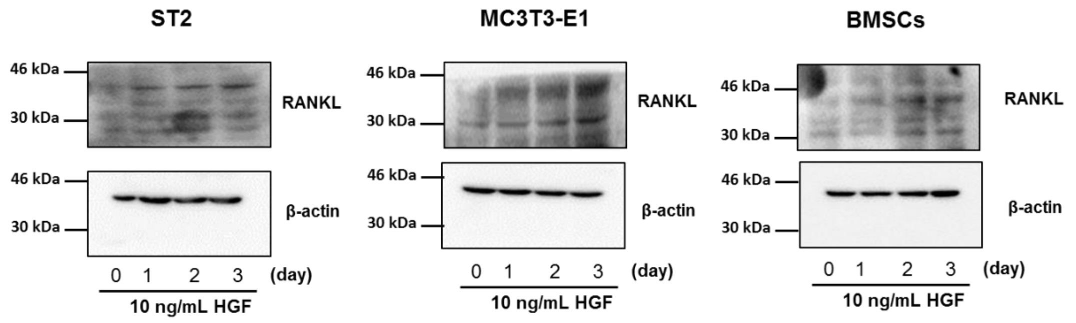

Figure 2A

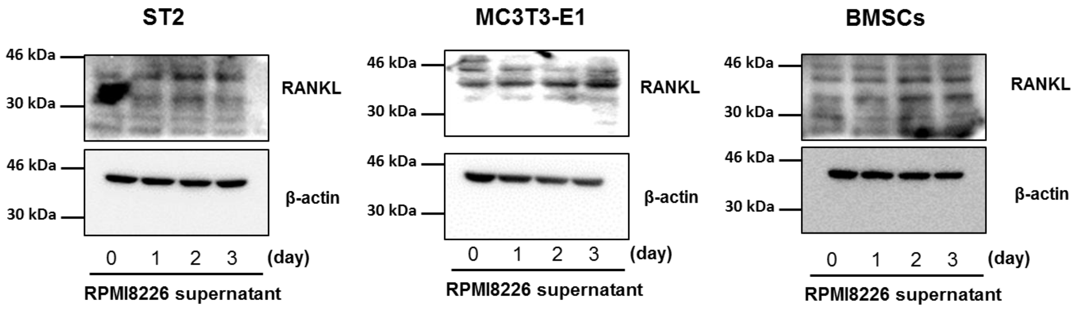

Figure 2C

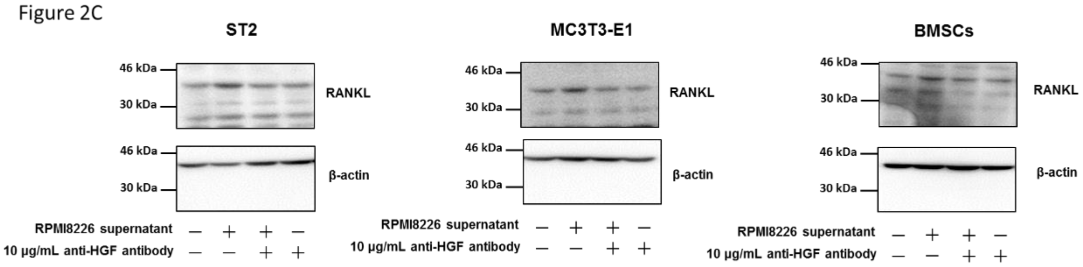

Figure 3A **ST2**

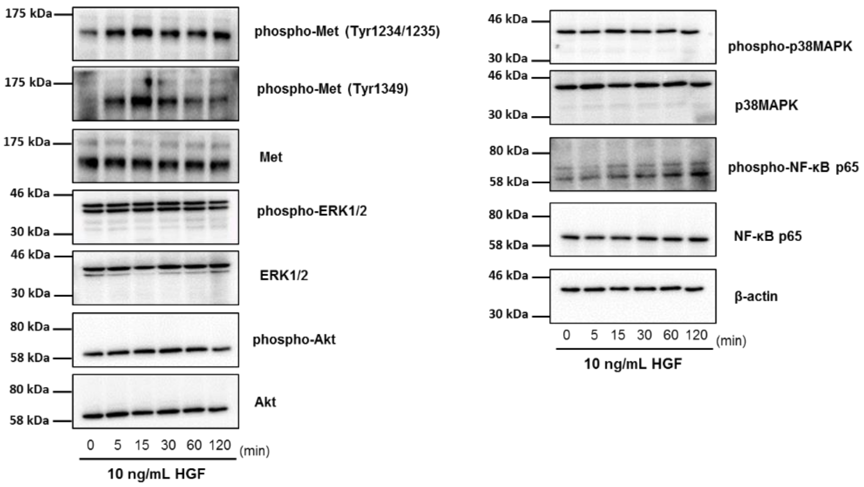

Figure 3A **MC3T3-E1**

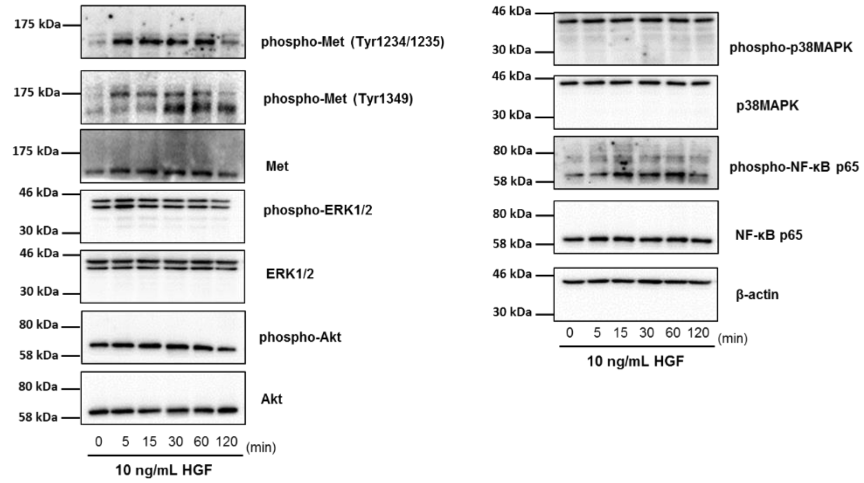

Figure 3A **BMSCs**

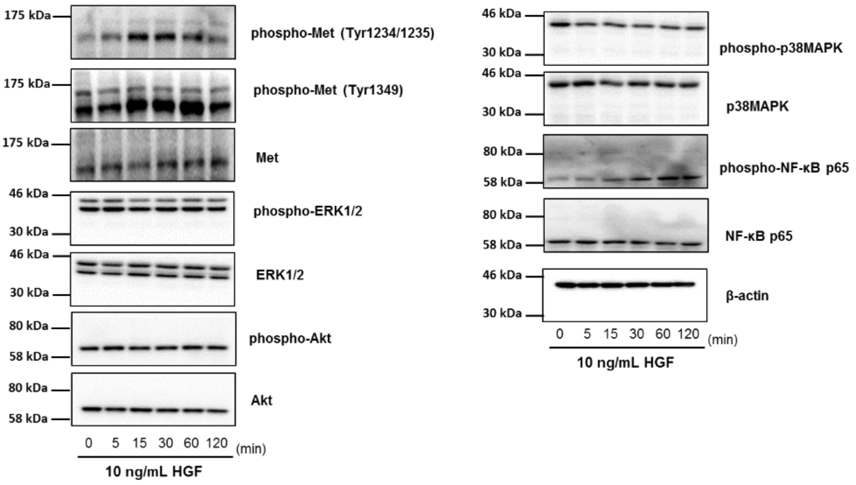

Figure 4C

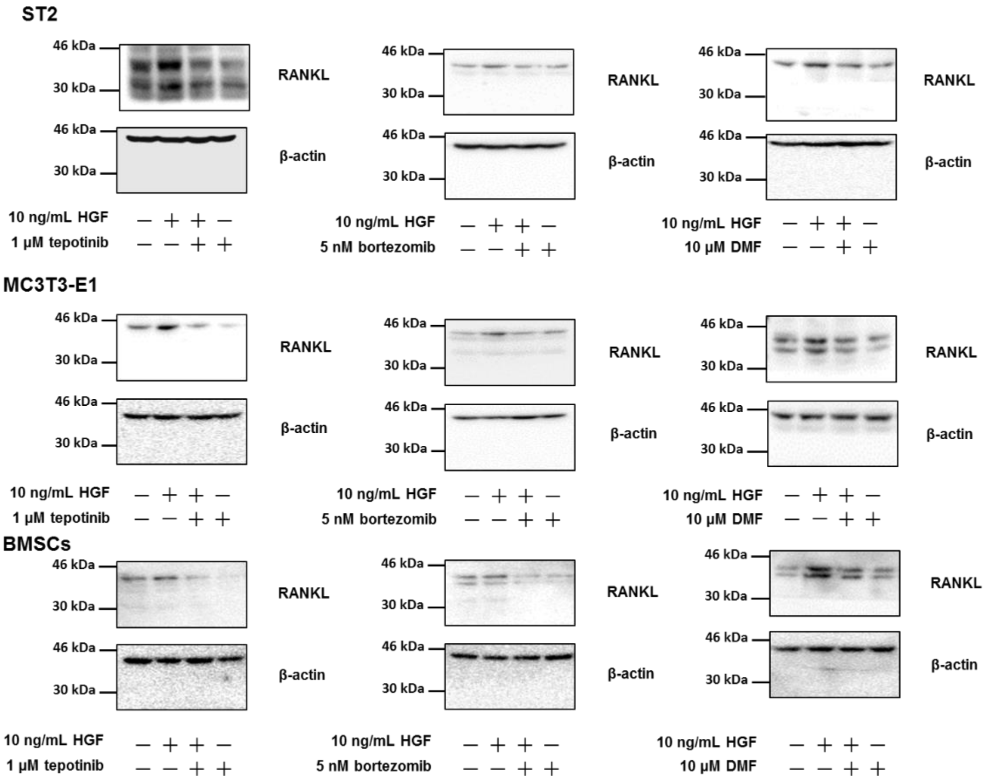
ST2
MC3T3-E1
BMSCs
46 kDa
30 kDa
RANKL
β-actin
10 ng/mL HGF
1 μM tepotinib
5 nM bortezomib
10 μM DMF
— + + —
— — + +

Figure S3

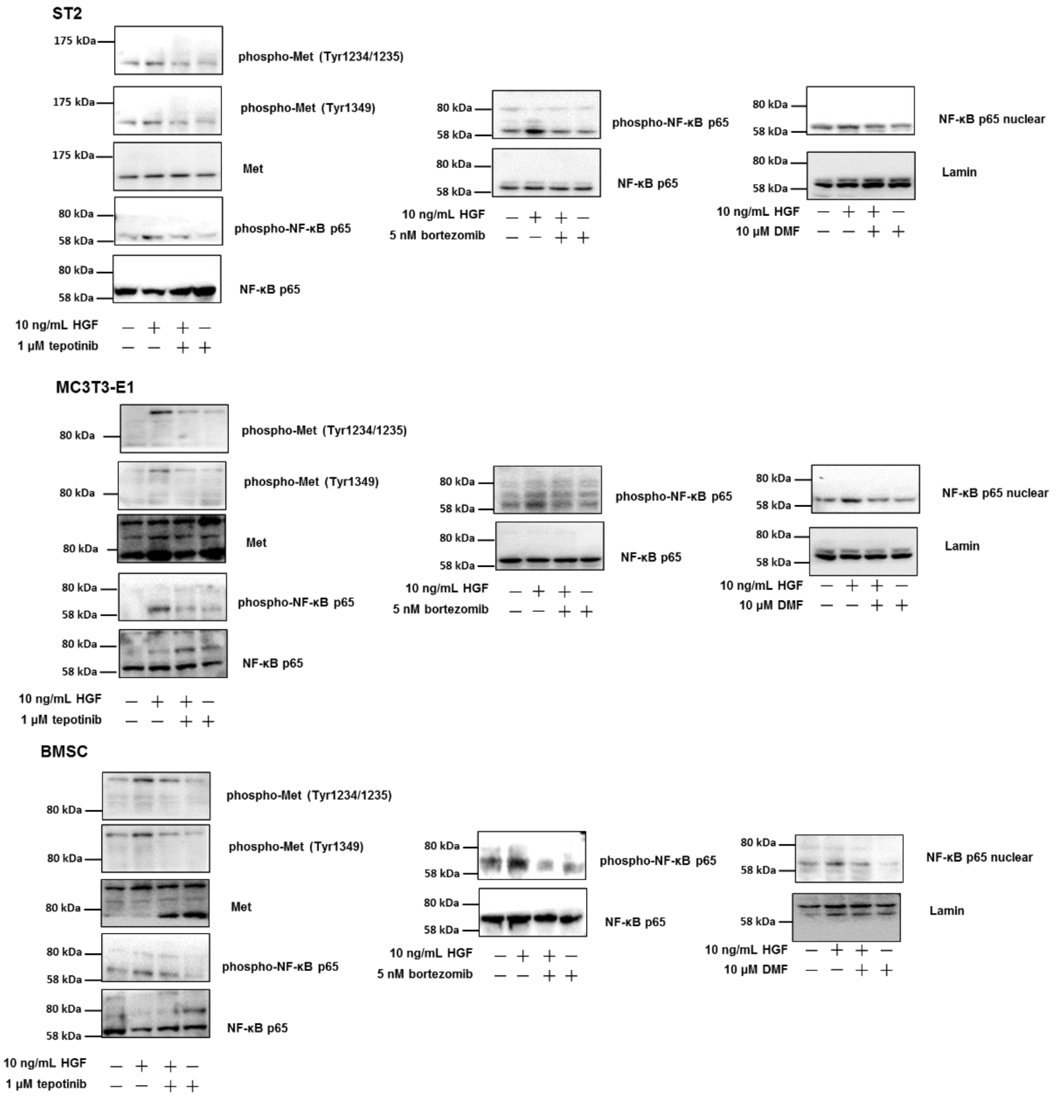
ST2
175 kDa
phospho-Met (Tyr1234/1235)
175 kDa
phospho-Met (Tyr1349)
175 kDa
Met
80 kDa
58 kDa
phospho-NF-κB p65
80 kDa
58 kDa
NF-κB p65
10 ng/mL HGF
1 μM tepotinib
80 kDa
58 kDa
phospho-NF-κB p65
80 kDa
58 kDa
NF-κB p65
10 ng/mL HGF
5 nM bortezomib
80 kDa
58 kDa
NF-κB p65 nuclear
80 kDa
58 kDa
Lamin
10 ng/mL HGF
10 μM DMF
MC3T3-E1
80 kDa
phospho-Met (Tyr1234/1235)
80 kDa
phospho-Met (Tyr1349)
80 kDa
Met
80 kDa
58 kDa
phospho-NF-κB p65
80 kDa
58 kDa
NF-κB p65
10 ng/mL HGF
1 μM tepotinib
80 kDa
58 kDa
phospho-NF-κB p65
80 kDa
58 kDa
NF-κB p65
10 ng/mL HGF
5 nM bortezomib
80 kDa
58 kDa
NF-κB p65 nuclear
80 kDa
58 kDa
Lamin
10 ng/mL HGF
10 μM DMF
BMSC
80 kDa
phospho-Met (Tyr1234/1235)
80 kDa
phospho-Met (Tyr1349)
80 kDa
Met
80 kDa
58 kDa
phospho-NF-κB p65
80 kDa
58 kDa
NF-κB p65
10 ng/mL HGF
1 μM tepotinib
80 kDa
58 kDa
phospho-NF-κB p65
80 kDa
58 kDa
NF-κB p65
10 ng/mL HGF
5 nM bortezomib
80 kDa
58 kDa
NF-κB p65 nuclear
58 kDa
Lamin
10 ng/mL HGF
10 μM DMF
